# Correlates of Depression in ART Adherence among Youths in Lilongwe, Malawi

**DOI:** 10.3390/tropicalmed9010002

**Published:** 2023-12-19

**Authors:** Mary Carolyne Msefula, Eric Umar

**Affiliations:** 1 Department of Health Sciences, School of Global and Public Health, Kamuzu University of Health Sciences (KUHeS), Blantyre P.O. Box 360, Malawi; eumar@kuhes.ac.mw; 2 Lighthouse Trust Malawi, Lilongwe P.O. Box 106, Malawi

**Keywords:** antiretroviral therapy, adherence, depression, youths, Malawi

## Abstract

Despite collaborative efforts to improve mental health services among youths living with HIV (YLHIVs) aged 15–24, evidence shows that many suffer from depression. We established the correlates of depression and how it affects ART adherence. Structured questionnaires, a Patient Health Questionnaire 9 depression scale assessment, in-depth interviews, and Electronic Medical Record reviews were conducted at the Lighthouse Trust Martin Preus Centre (MPC) ART clinic in Lilongwe from April 2021 to October 2022. A total of 303 YLHIVs aged 15–24 were on ART, and 7 key informants were recruited. Bivariate and multivariate logistic regression analyses were performed using STATA V14.1. A thematic content analysis was used for qualitative data. Forty-six per cent of recruits were male, and fifty-four per cent were female. Seventy-one per cent were aged 20–24, and twenty-nine per cent were aged 15–19. Twenty-three per cent of the YLHIVs had depression symptoms, of whom seventy-nine per cent were aged 20–24 years. Twenty-two per cent had an unsuppressed viral load (non-adherent). Sixty-seven per cent of non-adherent participants were aged 20–24. There was no factor associated with ART non-adherence. Source of income (*p* = 0.003), alcohol consumption (*p* = 0.010), and sexual behaviour (*p* = 0.014) were associated with depression. Sexual behaviour was statistically significantly associated with depression (*p* = 0.024. The themes were a lack of basic needs, a lack of privacy, psychological trauma, incomplete disclosure, a shortage of psychosocial providers, and a knowledge deficit of ART providers to screen for depression. This study shows that depression is considerably high among YLHIVs in Malawi and linked to ART non-adherence. Strengthening mental health training for providers and routinely screening YLHIVs for depression would help in the early identification and management of depression, thereby improving ART adherence.

## 1. Introduction

Despite the tremendous efforts in achieving the third 95 in the 95:95:95 United Nations Program on HIV/AIDs (UNAIDS) by 2030 strategy [1], youths on lifelong treatment are vulnerable to various mental health issues, which compromise their adherence to Antiretroviral Therapy (ART) [2]. In 2018, the number of young people living with HIV and AIDS was estimated at 4 million globally. It was further estimated that 1 million adolescents (15–19 years old) and 2.4 million young adults (20–24 years old) were living with HIV/AIDS in sub-Saharan Africa [3]. 

Countries in the sub-Saharan region are still struggling to meet the viral suppression rate as per the WHO target of 95:95:95. Significant factors for lower rates of viral suppression are a CD4 count >200 at baseline and switching to a second-line regimen [4]. The management of a high viral load includes intensive adherence counselling (IAC) sessions and a later switch of regimen after confirmed treatment failure. 

Malawi is among the countries in sub-Saharan Africa severely hit by HIV and AIDS, with an HIV prevalence rate of 3.3% among youths aged 15–24 [5]. Youths, particularly girls, are vulnerable to HIV infection because they lack negotiating powers over their bodies and are victims of various sexual violence experiences inflicted by their boyfriends, partners, and spouses [6]. An EGPAF audited report in Malawi established that 20% of adolescents had a high viral load, of whom, after IAC and repeated testing, 45% were still not virally suppressed, and 90% were switched to second-line ART [6]. However, adolescents aged 6–19 were noted to have continued high viral non-suppression. They were not switched to a new regimen after 48 weeks in Zimbabwe and Malawi [7], suggesting the mismanagement of a non-suppressed viral load.

MacCarthy et al., 2018 noted that most youths taking ART often go on drug holidays due to the burden of taking multiple medications daily [8], negative experiences following the self-disclosure of HIV status, a strong desire for secrecy, and restrictive school policies [9]. As such, privacy should be maintained in youths accessing ART services while on their school campuses to improve ART adherence and prevent HIV-related stigma. 

A lack of social support and engagement of health care service providers to adolescents and young adults (AYAs) aged 15–19 who have high-risk behaviour affects their ART adherence [8]. Providing close monitoring and support to these youths by strengthening youth-friendly services is paramount in ART services for youth empowerment and behaviour change. 

Strengthening social support and improving self-efficacy will enable viral suppression in 13–24-year-old youths [10]. Continuous monitoring, psychosocial support, and family support in ART management for youths will likely help to prevent non-adherence and improve quality of life. Likewise, peer support and other external environmental factors influence the creation of important networks for ART adherence in youths [8]. 

However, the same environmental and external relations may also negatively affect the behaviour of youths accessing ART services globally, which may also impact ART adherence. Similarly, ART adherence is mainly affected by the peer-to-peer stigma between those who are HIV-negative and YLHIVs, the side effects of ART, a lack of assistance, and forgetfulness [11]. Therefore, for adolescents accessing ART services, support from caregivers and peers fully equipped with knowledge of HIV care improves their ART adherence [11].

Mental and behavioural health challenges have a direct impact on all aspects of HIV prevention and treatment among YPLHIVs [12]. These challenges may be related to the biological impact of HIV diagnosis and lifelong treatment, which may include the psychosocial burdens of living with HIV and HIV-related social and environmental stressors [8]. It is noted that some youths who have lost their biological parents because of HIV have challenges with family support due to constant changes in guardianship [11]. Challenges associated with a lack of family support make youths stressed, thereby affecting their psychosocial behaviour and leading to conduct problems.

Conduct problems and depressive factors were found to contribute to non-adherence in youths who were using ART services in Rwanda [13]. The most presenting symptoms for conduct problems include suicidal thoughts and HIV risk-taking behaviour [10], and depressive symptoms include sadness, guilt, punishment, worthlessness, low energy, fatigue, irritability, depression, and suicidal thoughts [14].

According to Kim et al., 2015, 19% of adolescents aged 12–18 years in Malawi were depressed, with an association with few years of schooling and being bullied for taking ART medication [15]. However, untreated depression leads to the challenges of refusing ART and missing clinic appointments, thereby resulting in failure to achieve viral suppression [16]. 

The likelihood of achieving ART adherence was found to be 42% lower among those with depressive symptoms than those without [17]. The co-occurrence of depressive symptoms in YLHIVs and on ART affects their ART adherence [18]. A strong association was found between depression and adherence (PR: 2.06 (95%CI: 1.23–3.45)) in adolescents who had depressive symptoms and were sexually active, drinking alcohol, had lost both parents, and were not in school [18].

Likewise, a systematic review in Ethiopia noted that a high prevalence rate of depression was associated with discontinued education due to HIV/AIDS illness, poor treatment adherence, and a baseline high viral load [19].

It was further noted that adolescents who were depressed had few years of schooling and were being bullied for taking medication ART [15]. As such, their ART adherence was compromised, leading to unsuppressed viral particles. This is also prevalent in secondary school-going youths, as most lack privacy. 

HIV-related stigma was associated with increased psychological distress; physical and emotional/verbal abuse; low social support, isolation, and a feeling of rejection; and risky health behaviours, such as medication hiding, missing doses/non-adherence to ART, and poor health-seeking behaviours [10]. 

### 1.1. ART Services in Malawi

Antiretroviral Therapy (ART) services in Malawi are making strides in improving the quality of life of youths accessing HIV/AIDS services. Ministry of Health facilities and other partners such as the Lighthouse Trust and Baylor College of Medicine manage these adolescents’ services. These partners have Teen Clubs for YLHIVs, who are grouped and enrolled in ART programs depending on their age category, and follow-up care is one of the services provided. To ensure ART adherence, psychosocial, emotional health, and physical well-being care services are provided within the health facilities where enrolled Teen Club members access ART.

Several studies have been conducted in sub-Saharan Africa and Malawi on identifying high levels of mental health and the challenges faced by youths living with HIV/AIDS. In Malawi, there is limited literature on the correlates of depression in ART non-adherence among YLHIVs accessing ART services. Therefore, this study determined the ART adherence and prevalence of depression and measured the associated factors of depression in ART non-adherence among YLHIVs at MPC in Lilongwe.

### 1.2. Conceptual Framework 

Different researchers have employed various conceptual and theoretical frameworks to understand ART adherence, and among the theoretical frameworks used, the Social Action Theory (SAT) has often been utilised. Based on this theory, the factors associated with ART adherence include contextual factors, which are the environment, adolescence, mental health, baseline medical characteristics, and HIV clinic visits. The social and regulatory processes include social isolation, family rejection, stigma, disclosure, exploitation by older men, and limited interaction with clinic staff, and the health actions and outcomes include poor adherence and high-risk behaviours [20]. 

**Figure 1:**
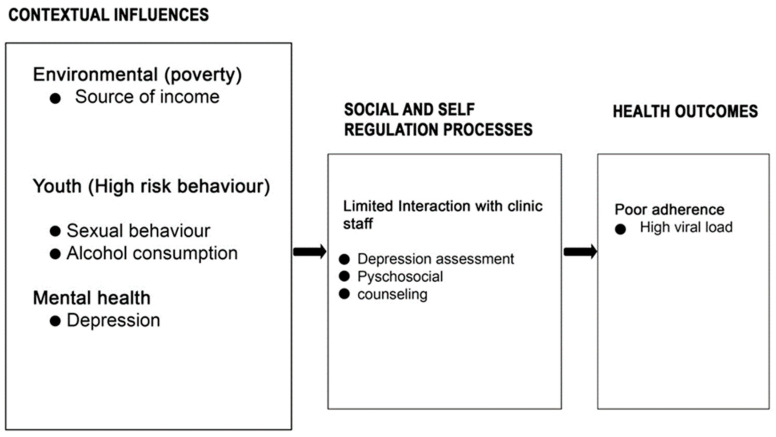
Conceptual framework Adapted from Seo et al. 2019 [21].

The framework assisted in the analysis of the findings, obtaining subsequent findings through thematic areas, which were influenced by the attributes in the framework. Figure 1, below, is an illustration of the adapted SAT framework. This study focused on contextual influence, precisely the source of income (poverty), sexual behaviour, alcohol consumption, depression assessment, psychosocial counselling, and the health outcome of ART adherence among youths in Lilongwe, Malawi. 

### 1.3. Justification 

MPC has vibrant, youth-friendly, competent, and skilled psychosocial counsellors and ART providers providing comprehensive HIV services to children, youths aged 15–24, and adults. Despite collaborative efforts in improving ART adherence among YLHIVs in Lilongwe, it was noted that some youths have been accessing psychosocial counselling services through either self-referral or on a provider-initiated basis due to ART non-adherence-related factors. Little was known as to whether there were associated factors of mental health (depression) in ART non-adherence amongst these youths aged 15–24 years. 

Therefore, this study set out to establish the correlates of depression in ART non-adherence among youths in Lilongwe. Specifically, it showed the prevalence of depression and explored associated factors in ART non-adherence among YLHIVs at MPC in Lilongwe, Malawi.

## 2. Materials and Methods

### 2.1. Study Design

This study used a cross-sectional mixed-method design with quantitative and qualitative approaches, with each providing equal weight (QUANT-QUAL), in youths who access ART services under the Lighthouse Trust at Martin Preus Centre (MPC).

### 2.2. Study Place

MPC was chosen because it is one of the biggest clinics in Malawi that provides comprehensive HIV/AIDS services to adults and children of all ages living with HIV in Lilongwe, Malawi. It serves over 25,000 clients, with ART provided to adults and children of all ages.

### 2.3. Study Population

For quantitative purposes, the study recruited youth participants aged 15–24 who were on ART during data collection at MPC. For qualitative purposes, youth participants aged 15–24, ART providers, mental health clinicians, mental health nurses, and psychosocial counsellors were recruited in the study.

YLHIVs aged 15–24 on ART at MPC in Lilongwe who had more than six months duration in ART care and viral load results within January 2021–January 2022 were recruited in the study.

ART providers, mental health clinicians, mental health nurses, and psychosocial counsellors who had worked with youths at MPC for more than a year were recruited in the study.

### 2.4. Sample Size and Sampling

Using the formula [*n* = z^2^P(1 − P)/m^2^], a sample size of 310 youths was supposed to be recruited for the survey component of the study. The number of study participants was determined and calculated based on the total number of youths aged 15–24 who accessed psychosocial counselling services at MPC due to mental health issues and ART non-adherence-related factors. However, 303 (98%) youth participants were recruited out of the 310 proposed sample size because the other 7 (2%) youths were unavailable for interviews, as they were in boarding secondary schools. Convenient sampling was employed to recruit these participants. Youths attending the health facility were recruited on the day that they came to seek various services. 

For the qualitative component of the study, a total of 19 youths were interviewed. The number was determined via data saturation. After interviewing 14 youths, no new information emerged, with the subsequent five interviews signalling saturation. Seven health service providers were interviewed. 

The providers were purposively recruited because they have a deeper understanding and knowledge of and skills in depression assessment and ART non-adherence counselling and management. Conveniently, the providers and 303 youths were recruited by taking advantage of their presence on a particular working day, when ART services were being provided to the youths.

### 2.5. Data Collection 

Data collection took 30 days. This was due to variations in the youth group’s appointment dates, which allowed the principal investigator enough time to collect data and ensure that all eligible participants participated in the study.

#### 2.5.1. Quantitative Method

Data were collected using the Patient Health Questionnaire (PHQ 9) checklist scale for depression assessment. PHQ 9 is a depression screening checklist that helps in assessing symptoms and categorising their severity amongst clients, ranging from mild, moderate, and severe depending on the scores of the symptoms.

Secondary data were extracted from Electronic Medical Records (EMRs) to assess the 303 youths’ ART adherence indicated by a high viral load from January 2021 to January 2022. Later, the collected data were examined to determine the correlates of depression (predictor variables) in ART adherence (outcome variable) by comparing the proportion of patients with poor ART adherence among the group with depression.

#### 2.5.2. Qualitative Method

Data were collected using a semi-structured interview guide on in-depth interviews (IDIs) to determine the in-depth knowledge of youth participants, mental health clinicians, mental health nurses, psychosocial counsellors, and ART providers on factors influencing depression in ART non-adherence amongst youths. The interview guides were developed based on the concepts of an adapted SAT framework. The IDIs were guided by open-ended questions, which were audio-recorded and backed with field notes.

### 2.6. Data Analysis

A thematic content analysis through the organisation of questions and themes was conducted, and an adapted Social Action Theory (SAT) was used to determine the associated factors underlining depression and ART non-adherence [21].

ART adherence in this study was defined by measuring the load of viral particles in the HIV-infected youths annually. A suppressed viral load was determined if readings were below 200 copies, and an unsuppressed viral load was determined at any figure above 200 copies; hence, youths were regarded as prone to treatment failure.

STATA V.14.1 software was used to analyse the quantitative data and then mined to see the correlation between depression and ART non-adherence in youths [15,16,17,18,19,20,21,22,23,24] accessing HIV services. The multivariable logistic regression model was used to analyse all variables with a *p*-value of less than 0.05 to determine their significance. Then, the Pearson chi-square test was used to assess the correlation between depression and ART adherence by computing odds. The convergent parallel design was used during the data analysis to ensure that the quantitative and qualitative methods were weighed equally, analysing both methods independently and interpreting the results together [23].

### 2.7. Ethical Considerations

Ethical approval was sought from the College of Medicine Research Committee (COMREC) to prevent the violation of human rights during the study period (P.08/21/3372 dated 15 September 2021). We sought permission from the Lighthouse Trust MPC clinic since it was where the participants were gathered from, and participants’ informed consent, key informants’ consent, and parental consent for adolescents aged 15–17 years were provided before data collection.

## 3. Results

The results of this study were split into quantitative and qualitative results. This is because of the nature of the research question answers, which were better presented separately and integrated into the Discussion Section.

### 3.1. Quantitative Findings

#### 3.1.1. Social and Demographic Characteristics of Study Participants

The characteristics of the youth participants *n* = 303 were as follows: 29% were adolescents [15,16,17,18,19], and 71% were youths [20,21,22,23,24]; 90% were Christians; interestingly, 11% of participants reported to have used alcohol; 16% of participants had a means of sourcing income for their basic needs; and 25% of participants reported having at least one sexual partner (see Table 1 below). 

#### 3.1.2. ART Adherence (Viral Load) Characteristics

Viral load was used to measure ART adherence, dichotomised into suppressed for those with less than 200 copies per ml and unsuppressed for those with more than 200 copies per ml. The study found that 67 (22%) had an unsuppressed viral load, of whom 67% were from the 20–24 youth group. The study further noted that 75% of the participants who depended on their parents as a source of income had an unsuppressed viral load, and more than half (63%) of those with an unsuppressed viral load had secondary education (see Table 2 below). 

#### 3.1.3. Depression among the Youths

Depression was one of the independent variables explored in the study. The study noted that out of *n* = 303, 71 (23%) had depression, almost half (51.71%) had mild depression, 26% had moderate depression, and 1% had severe depression. The study further noted that 79% of depression cases were from the 20–24 age category, and above half (56%) depended on their parents for their source of income (see Table 3 below).

#### 3.1.4. Bivariate Analysis 

The study noted that the source of income (*p*-value 0.003), alcohol consumption (0.010), and sexual behaviour (0.014) were factors associated with depression in YLHIVs. In terms of specific categories, depression symptoms were more likely to occur in the 20–24 age category. However, there were no statistical associations between ART adherence and the following variables: age, sex, marital status, education, smoking, and alcohol consumption.

#### 3.1.5. Multivariate Logistic Regression Results 

To adjust for potential confounders, multivariate logistic regression was performed among the depression variables that had a p-value of less than 0.050 after being analysed. The cut-off <0.050 was set to determine the highly significant variables, and the following depression variables all had *p*-values of less than 0.05: source of income (Pearson chi2 = 19.5622, *p* = 0.003), alcohol consumption (Pearson chi2 = 11.3206, *p* = 0.010), and sexual behaviour (Pearson chi2 = 20.7351, *p* = 0.014). The study found that the youth participants with a single sexual partner had a two times increased chance of suffering from depression than those without a sexual partner. Sexual behaviour was statistically significant in causing depression, with a *p*-value of 0.024 (see Table 4 below). 

### 3.2. Qualitative Findings

The study attempted to assess ART adherence amongst the youths and explored factors leading to depression among the youths in Lilongwe. 

#### 3.2.1. Explanatory Models for ART Non-Adherence amongst the Youths 

The themes perceived following the in-depth interviews with the youth participants were as follows: a lack of basic needs, a lack of privacy, and psychological trauma.

##### Lack of Basic Needs

Basic needs are important and required for survival, and most youths who are HIV-positive have more challenges in obtaining basic needs, such as food, clothing, and school fees; hence, dependency syndrome occurs. The respondents acknowledged that lacking basic needs such as food makes it challenging to adhere to ART management.

“*Sometimes I am always busy doing peace work to earn money for my daily living, hence forgetting to take my daily medications*” (Youth).

This is worsened by orphanhood due to multiple changes in guardianship, hence some basic needs being unmet. “The inability to meet their basic needs makes them start engaging in sexual behaviours in search of money, leading to early pregnancies, sexually transmitted infections, and early marriages” (Psychosocial Counsellor).

##### Lack of Privacy 

For ART adherence, the respondents stressed the need to ensure privacy to youths on lifelong treatment to allow them to feel secure and be socially included in daily activities at school and home.

“*The youths who are in boarding facilities are challenged with privacy as they want to hide that they are on ARV’s and others are in an exploratory phase of different social behaviours, for example, smoking, alcohol and drug use influenced by their peers and later makes them forget taking ARV’s or stops taking ART*” (ART Clinician).

This was concurred by a youth:

“*Because ARVs are unlike other drugs, they need strict privacy so that people will not discriminate against you. For instance, in the last six months of ART appointments, I have missed doses three times since I was at school, and there were many friends around me which made me fail to take up my ARV as prescribed*” (Youth).

##### Psychological Trauma

Some youth respondents acknowledged struggling in adhering to ART because of the verbal abuse that they receive in their homes from their relations and guardians, who subject them to discrimination.

“*My relation once disclosed my status to others by speaking bad words such as look at this one. He is HIV positive, and this honestly made me think of stopping taking ARV’s so that I die and follow my parents, and sometimes it’s because of self-disclosure due to angriness that you have tested positive*” (Youth)

### 3.3. Explanatory Models for Depression among the youths

Following an in-depth interview with 7 key informants on causes of depression, the following themes were identified: incomplete disclosure, the shortage of psychosocial providers, and the knowledge deficit of ART providers.

#### 3.3.1. Incomplete HIV Status Disclosure 

The respondents acknowledged having partial knowledge or wrong information on why they are the only ones taking ART out of their siblings, and this is exacerbated by orphanhood.

“*They tend to ask why they are the only ones with HIV in their house and why their parents failed to disclose to them when they were alive, which triggers confusion*” (Psychosocial counsellor).

#### 3.3.2. Shortage of Psychosocial Providers

The respondents acknowledged that many youths fail to acquire psychosocial counselling services in a timely manner, and their conditions progress faster to mild depressive symptoms because of untimely access to psychosocial counselling services due to shortages of psychosocial providers.

“*Only those youths presenting with depression symptoms are the ones given a chance to access the services on the adolescent clinic days because of shortage of psychosocial providers which even compromise on the quality of counselling services we do provide to the youths*” (Psychosocial counsellor).

#### 3.3.3. Knowledge Deficit of ART Providers to Screen Depression 

ART providers reported that updated knowledge is critical for the management of youths on ART, as it helps to screen youths for depression and manage them in a timely manner, hence affecting ART adherence.

“*Other ART providers are not knowledgeable and skilled on youth’s depression assessments, which compromise the quality of care they receive at the clinic*” (Nurse ART provider).

## 4. Discussion

This study aimed to establish the correlates of depression with ART adherence amongst youths aged 15–24 in the central region of Malawi. Youths living with HIV face various issues that affect their adherence to ART. The results were guided by the use of Social Action Theory, which states several potential factors that could lead to poor adherence and high-risk behaviours in adolescents. 

In a sample population where females were in the majority, the findings show that 11% of the youths reported alcohol use. Alcohol use is one of the significant health-related risk behaviours of youths, increasing their vulnerability to mental health conditions [2], which, later on, may make them miss ART doses, thus compromising their adherence. In the current study, factors like source of income, alcohol consumption, and sexual behaviour were mentioned in qualitative interviews as factors that could lead to ART non-adherence. However, these factors were not statistically significantly associated with ART adherence despite being mentioned in the qualitative interviews as potential factors leading to non-adherence. This is not in agreement with Kim et al., 2015, who noted that drinking alcohol in the past month was independently associated with missing doses/ART non-adherence [15]. Other factors not related to ART non-adherence were sexual behaviour and source of income. These factors may not have led to ART non-adherence because the majority of these youths receive their medicines from a facility that supports their medication adherence through Teen Clubs, hence increasing their self-efficacy in medication adherence. Support and self-efficacy reinforce ART adherence in similar contexts and age groups (REF. UMAR).

Surprisingly, three-quarters (75%) of those with an unsuppressed viral load were YLHIVs aged 20–24 who depended on their parents as a source of income to meet their basic needs. When these basics are not fully met in youths who are on ART, they may start engaging in risky behaviours, such as prostitution, alcohol, drug substance abuse, and early marriage [11, 13]. These behaviours may influence ART adherence, as some may start missing their doses due to stress. This is in line with the findings from a Ugandan study, which noted that economic stressors were factors associated with depressive symptoms and missed ART doses, which affected ART adherence in people living with HIV [24]. However, the study did not determine whether the parents could fully meet the basic needs of YLHIVs.

Above half (63%) of those with an unsuppressed viral load in secondary school education reported a lack of privacy as their biggest challenge. This has been proven to affect ART adherence on some days. They skip their drugs out of fear, negative thoughts, and self-devaluation of being discriminated against and excluded by their peers [25]. McCarthy, 2018, concurred that youths at secondary and tertiary education levels have disrupted ART adherence due to a lack of privacy [8]. 

Therefore, if youths are to comply with ART adherence while on school campus, privacy has to be promoted; failure to address privacy challenges may lead to HIV-related stigma and discrimination. Hence, others may opt to default or stop taking treatment, which may lead to ART non-adherence outcomes, such as an unsuppressed viral load [26].

Incomplete disclosure of youths’ HIV status due to orphanhood also contributes to ART non-adherence, as some youths ask why they are the only ones taking ART, and not everyone at home is aware of the adolescent’s HIV status, which affects ART adherence [27]. These youths face challenges in decision making, as they are prone to multiple changes in guardianship and, hence, excluded from important decisions in their lives [28]. Dependency syndrome has been shown to put YLHIVs at high risk of behaviours such as multiple sexual behaviours, leading to sexually transmitted infections and early pregnancies, in search of social support for basic daily needs [29]. It was further noted that participants who have moderate depression are nearly two times more likely to experience risky sexual behaviour than students with no depression symptoms [29]. This may lead to an unsuppressed viral load among youths, especially those in secondary school. 

Almost one-quarter of the participants had depression with either mild or moderate symptoms, and over half depended directly on their parents as a source of income to provide their basic needs. YPLHIs were very likely to suffer from depressive symptoms when their basic needs were not met, later affecting ART adherence. This was indicated by the result being below the cut-off of the significance line (0.003), and it was concluded that it statistically significantly influenced ART adherence. However, it was challenging to quantify their income levels, and this study did not include parents/guardians on the list of participants to examine their source of income and other related factors. 

The lack of knowledge of ART providers, the shortage of psychosocial counsellors, and untimely psychosocial support provided to YLHIVs contribute to a delay in the early diagnosis of depressive symptoms. This may be influenced by the knowledge levels of the providers, which compromise screening processes for depression, which could be treated at an earlier stage. Later on, mild depression may rapidly progress to severe depression. This may imply the need to improve ART adherence among youths. A scoping review noted that individual counselling, support groups, family-centred services, and treatment supporters could improve ART adherence in YLHIVs [30]. Likewise, strengthening social support helps in enhancing self-efficacy among youths living with HIV [10].

## 5. Conclusions

Depression is considerably high among YLHIVs receiving ART in Lilongwe and is linked to non-ART adherence. Hence, youths living with HIV need close monitoring and continuous psychosocial support for the early detection of depressive symptoms if we are to improve ART adherence.

Providing holistic mental health services through depression screening, continuous psychosocial support to youths with depressive symptoms, and access to ART services in Malawi would improve ART adherence.

## Figures and Tables

**Table 1:** Social and demographic characteristics of study participants.

Characteristic	Frequency (*n* = 303)
Sex	
Male	139 (46%)
Female	164 (54%)
Total	303 (100%)
Marital status	
Never married	278 (92%)
Married	22 (7%)
Divorced	3 (1%)
Widow	0
Total	303 (100%)
Religion	
Christianity	273 (90%)
Islam	30 (10%)
Total	303 (100%)
Education	
None	1 (0.3%)
Primary	100 (33%)
Secondary	192 (63%)
Tertiary	10 (3%)
Total	303 (100%)
Source of income	
Self	45 (15%)
Parents	223 (74%)
Guardians	35 (12%)
Total	303 (100%)
Alcohol consumption	
No	271 (89%)
Yes	32 (11%)
Total	303 (100%)
Sexual behaviour	
None	271 (89%)
One partner	75 (25%)
Two partners	14 (5%)
>Two partners	7 (2%)
Total	303 (100%)

**Table 2:** ART adherence (viral load) among the youths.

Characteristics	Suppressed (*n* = 236) *n* (%)	Unsuppressed (*n* = 67) *n* (%)	*p*-Value
Age			
15–19	65 (28%)	22 (33%)	
20–24	171 (72%)	45 (67%)	
Total	236 (100%)	67 (100%)	0.398
Sex			
Male	110 (47%)	28 (42%)	
Female	126 (53%)	39 (58%)	
Total	236 (100%)	67 (100%)	0.485
Marital status			
Never married	226 (96%)	63 (94%)	
Married	8 (3%)	3 (4%)	
Divorced	2 (0.8%)	1 (2%)	
Total	236 (100%)	67 (100%)	0.816
Religion			
Christianity	215 (91%)	58 (87%)	
Islam	21 (9%)	9 (13%)	
Total	236 (100%)	67 (100%)	0.273
Education			
None	0	1 (2%)	
Primary	78(33%)	22 (33%)	
Secondary	150(64%)	24 (36%)	
Tertiary	8((3%)	2 (3%)	
0.314	236(100%)	67 (100%)	
Source of income			
Self	35 (15%)	10 (15%)	
Parents	173 (73%)	50 (78%)	
Guardians	28 (12%)	7 (10%)	
Total	236 (100%)	67 (100%)	0.950
Alcohol consumption			
No	212 (90%)	59 (88%)	
Yes	24 (10%)	8 (12%)	
Total	236 (100%)	67 (100%)	0.677
Sexual behaviour			
None	159 (67%)	48 (72%)	
One partner	59 (25%)	16 (24%)	
Two partners	13 (6%)	1 (1%)	
>two partners	5 (2%)	2 (3%)	
Total	236 (100%)	67(100%	0.541

**Table 3:** Depression characteristics.

Characteristics	None (*n* = 232)	Mild (*n* = 51)	Moderate (*n* = 19)	Severe (*n* = 1)	*p*-Value
Age					
15–19	72 (31%)	10 (20%)	5 (26%)	0	
20–24	160 (69%)	41 (80%)	14 (74%)	1 (100%)	
Total	232 (100)	51 (100)	19 (100)	1 (100%)	0.372
Sex					
Male	103 (44%)	27 (53%)	8 (42%)	0	
Female	129 (56%)	24 (47%)	11 (58%)	1 (100%)	
Total	232 (100)	51 (100)	19 (100)	1 (100)	0.537
Marital status					
Never married	220 (95%)	49 (96%)	19 (100%)	1 (100%)	
Married	9 (4%)	2 (4%)	0	0	
Divorced	3 (1%)	0	0	0	
Total	232 (100%)	51 (100%)	19 (100%)	1 (100%)	0.941
Religion					
Christianity	210 (91%)	44 (86%)	18 (95%)	1 (100%)	
Islam	22 (9%)	7(14%)	1 (5%)	0	
Total	232 (100%)	51 (100%)	19 (100%)	1 (100%)	0.694
Education					
None	1 (0.4%)	0	0	0	
Primary	77 (33%)	16 (31%)	7 (37%)	0	
Secondary	145 (63%)	35 (69%)	11 (58%)	1 (100%)	
Tertiary	9 (4%)	0	1 (5%)	0	
Total	232 (100%)	51 (100%)	19 (100%)	1 (100%)	0.942
Source of income					
Self	31 (13%)	9 (18%)	5 (26%)	0	
Parents	183 (79%)	29 (57%)	10 (53%)	1 (100%)	
Guardians	18 (8%)	13 (25%)	4 (21%)	0	
Total	232 (100%)	51 (100%)	19 (100%)	1 (100%)	0.003
Alcohol consumption					
No	211 (91%)	45 (88%)	15 (79%)	0	
Yes	21 (9%)	6 (12%)	4 (21%)	1 (100%)	
Total	232 (100%)	51 (100%)	19 (100%)	1 (100%)	0.010
Sexual behaviour					
None	169 (73%)	26 (51%)	12 (63%)	0	
One partner	51 (22%)	17 (33%)	6 (32%)	19 (100%)	
Two partners	7 (3%)	7 (14%)	0	0	
>Two partners	5 (2%)	1 (2%)	1 (5%)	1 (100%)	
Total	232 (100%)	51 (100%)	19 (100%)	1 (100%)	0.014

NOTE *p* = *p*-value. Any *p* < 0.005 = highly significant, *p* > 0.005 = not significant.

**Table 4:** Multivariate logistic regression.

Variable	Odds Ratio	*p* > [z]	95% Confidence Level	
**Source of income**				
Self (reference)	1			
Parents	0.5975	0.188	0.2777	1.2855
Guardians	2.3149	0.08	0.904	5.9271
**Alcohol consumption**				
No (reference)	1			
Yes	1.8993	0.142	0.8065	4.4728
**Sexual behaviour**				
None (reference)	1			
One partner	2.01314	0.024	1.0976	3.7596
Two partners	2.1197	0.172	0.7211	6.2304
More than two	1.0967	0.922	0.1729	6.9553

## Data Availability

Data presented in the study are unavailable due to privacy in vulnerable populations.
